# Incidence of and Experiences with Abortion Attempts in Soweto, South Africa: Respondent-Driven Sampling Study

**DOI:** 10.2196/38045

**Published:** 2022-12-08

**Authors:** Caitlin Gerdts, Ruvani T Jayaweera, Relebohile Motana, Tshegofatso Bessenaar, Paul Wesson

**Affiliations:** 1 Ibis Reproductive Health Oakland, CA United States; 2 Ibis Reproductive Health Johanesburg South Africa; 3 Department of Epidemiology and Biostatistics School of Medicine University of California, San Francisco San Francisco, CA United States

**Keywords:** induced abortion, respondent-driven sampling, self-managed abortion, abortion incidence

## Abstract

**Background:**

Estimation of abortion incidence, particularly in settings where most abortions occur outside of health facility settings, is critical for understanding information gaps and service delivery needs in different settings. However, the existing methods for measuring out-of-facility abortion incidence are plagued with methodological challenges. Respondent-driven sampling (RDS) may offer a methodological improvement in the estimation of abortion incidence.

**Objective:**

In this study, we tested the feasibility of using RDS to recruit participants into a study about abortion and estimated the proportion of people who ever attempted abortion as well as 1-year and 5-year incidence of abortion (both in-facility and out-of-facility settings) among women of reproductive age in Soweto, South Africa.

**Methods:**

Participants were eligible if they identified as a woman; were aged between 15 and 49 years; spoke English, Tswana, isiZulu, Sotho, or Xhosa; and lived in Soweto. Working with community partners, we identified 11 seeds who were provided with coupons to refer eligible peers to the study. Upon arrival at the study site, the recruits completed an interviewer-administered questionnaire that solicited information about demographic characteristics, social network composition, health behaviors, sexual history, pregnancy history, and experience with abortion; recruits also received 3 recruitment coupons. Recruitment was tracked using coupon numbering. We used the RDS-II estimator to estimate the population proportions of demographic characteristics and our primary outcome, the proportion of people who ever attempted abortion.

**Results:**

Between April 4, 2018, and December 17, 2018, 849 eligible participants were recruited into the study. The estimated proportion of people who ever attempted abortion was 12.1% (95% CI 9.7%-14.4%). A total of 7.1% (95% CI 5.4%-8.9%) reported a facility-based abortion, and 4.4% (95% CI 3.0%-5.8%) reported an out-of-facility abortion.

**Conclusions:**

The estimated proportion of people who ever attempted abortion of 12% (102/849) in our study likely represents a substantial underestimation of the actual proportion of abortion attempts among this study population—representing a failure of the RDS method to generate more reliable estimates of abortion incidence in our study. We caution against the use of RDS to measure the incidence of abortion because of persistent concerns with underreporting but consider potential alternative applications of RDS with respect to the study of abortion.

## Introduction

In contexts where abortion is legally restricted or where other barriers exist, abortion commonly occurs without the involvement of the formal health care sector [1,2] using a variety of methods ranging from safe, World Health Organization–recommended medications [3] to the ingestion of harmful substances. Forthwith, we will refer to all such abortions as *out-of-facility* abortions. The most recent global estimates suggest that approximately 45% of abortions worldwide from 2010 to 2014 took place outside a health care facility, whereas in specific settings, out-of-facility abortions comprised 70% to 80% of all abortions [4]. Researchers have studied out-of-facility abortion for decades [5]; however, the existing data sources on out-of-facility abortions often suffer from selection bias, misclassification, and underreporting and have led to documented underestimates of abortion incidence [6] and unreliable data on the characteristics of abortion seekers and outcomes of abortion in such contexts [7,8].

Multiple innovations in the estimation of out-of-facility abortion incidence have been tested in recent years, none of which have emerged as a reliable gold standard [9-12]. As out-of-facility abortion becomes an increasingly common and supported model for abortion around the globe, there is a pressing need for new and innovative research methods that can more accurately measure the prevalence, incidence, and characteristics of out-of-facility abortions.

Respondent-driven sampling (RDS), a sampling methodology that relies upon social networks to identify populations engaging in stigmatized, illicit, or otherwise hidden behaviors, may offer a previously untested alternative to measuring out-of-facility abortion. Studies that use RDS begin with a small nonrandom sample of point people (ie, seeds) within social networks engaging in hidden or stigmatized behaviors, who are interviewed and provided with referral coupons to recruit others within the same social network (ie, the target population). RDS has been used to estimate the prevalence of sensitive and illegal behaviors among hidden populations such as people who inject drugs, sex workers, and men who have sex with men; and relies upon social networks to identify populations for whom no valid sampling frame exists [13-19]. To account for potential selection bias because of peer-to-peer recruitment, RDS inference methods inversely weight participants according to their social network size. Inference from the RDS data additionally requires several assumptions around the recruitment process. These assumptions include the following: all relationships between recruiters and their recruits are reciprocal, the composition of the final sample is independent of the composition of the initial seeds, sampling mimics sampling with replacement, participants can accurately estimate their degree, and recruiters randomly recruit from within their social network [20]. As RDS studies are typically conducted among populations with no valid sampling frame, an empirical assessment of whether RDS yields a representative sample is impossible in most contexts. Studies that have been able to assess these assumptions or compare RDS estimates with population estimates have found that although RDS generally yields a representative sample, RDS estimators often fail to reduce bias when it does exist, and recruitment assumptions are often not met [20-22]. However, RDS does allows for the recruitment of individuals who would not likely be identified or reached via traditional sampling methods.

RDS has never before been implemented to study abortion, and although RDS has most commonly been used to measure outcomes among a stigmatized population, this study is, to our knowledge, the first example of using RDS to measure abortion (a stigmatized outcome) among a general population. We hypothesized that RDS could be well suited to the measurement of out-of-facility abortion for a range of reasons. First, population-representative surveys, such as household surveys, may systematically exclude young women, women living in informal settlements, or female refugees. Furthermore, traditional direct survey techniques often result in participants underreporting their abortion experiences [1,2]. RDS has the potential to reach a broader population than the existing methods for abortion measurement, and the process of being recruited into the study by someone known to the participants may generate trust between the participant-recruiter and the researcher and encourage the disclosure of sensitive experiences.

In the Republic of South Africa, the Choice on Termination of Pregnancy Act, passed in 1996, allows for the legal termination of pregnancy on request up to 12-week gestation; under socioeconomic, incest, rape, and medical grounds from 12 to 20 weeks; and to save a pregnant person’s life after 20 weeks. Abortion services are provided free of charge in the public sector. However, barriers to abortion access in South Africa remain: a shortage of trained and willing providers [23] and a lack of dedicated facilities in which to perform abortions [24] can result in waiting lists that cause delays for abortion seekers, often beyond the legal gestational limit [25,26]. The most recent global estimates of abortion incidence, from 2015 to 2019, suggest an annual average of 30 abortions per 1000 women of reproductive age [27], and no reliable estimates exist for the proportion of abortions that occur within or outside facility settings in South Africa. Although out-of-facility abortions are widely known to occur in South Africa [24,28,29], their prevalence, safety, and effectiveness remain unknown. Although some data exist on people’s experiences with out-of-facility abortion in South Africa [30,31], reliable information about the prevalence of and people’s experiences with abortions that occur outside of the formal health system is needed both to inform improvements in abortion services, as well as to inform the development of resources about abortion that meet the needs and experiences of people in South Africa

In this study, we tested the feasibility of using RDS to sample participants and estimate the proportion of people who have ever attempted abortion—both those that occurred in-facility settings and those that occurred outside-of-facility settings—among the women of reproductive age in Soweto, South Africa. To assess feasibility, we considered (1) our ability to reach the proposed sample size, (2) whether RDS inference methods generated a sample similar to the source population, and (3) whether abortion was underreported.

## Methods

### Recruitment and Procedures

This study was conducted in Soweto, South Africa, from April 2018 to December 2018. Soweto is a large township within the city of Johannesburg with a total population of 1.3 million [32]. We used RDS, a well-established sampling method for populations for which there is no sampling frame, to calculate the proportion of people who have ever attempted abortion as well as the 1-year and 5-year cumulative incidence of abortion among women aged 15 to 49 years in Soweto.

With the assistance of well-known community-based organizations that provided a range of services, including, but not limited to, reproductive health services in Soweto, we recruited 11 women to serve as our initial seeds for RDS recruitment. These women were of various ages, income levels, and sexual and reproductive health experiences, including those with prior abortion. In accordance with the RDS methodology, seeds were members of the target population and purposively selected by our research team to initiate recruitment chains. We selected 2 study sites based on recommendations from our community-based organization partners about accessibility within the community and considerations of confidentiality—specifically, locations where people commonly gather or seek a range of services not specific to reproductive health. Seeds presented at one of the 2 study sites and completed an interviewer-administered questionnaire on their experiences with abortion. After completing the questionnaire, seeds were given 3 coupons to refer eligible peers to the study. Recruitment coupons contained information about the eligibility criteria, instructions on how to schedule an interview, and information about the study incentives. Potential participants contacted the study phone number via SMS text message or call and answered a short screening questionnaire to assess their eligibility. Participants were eligible if they identified as a woman; were aged between 15 and 49 years; spoke English, Tswana, isiZulu, Sotho, or Xhosa; and lived in Soweto. It is important to acknowledge that people of all genders have and need for abortions, and not all of them identify as women; some identify as men or another gender, and some people who identify as women do not have the capacity to carry a pregnancy. In the context of this study, we recruited people who identified as “women” and have referred to the study population accordingly throughout this paper.Eligible participants were invited to schedule an in-person interview at one of the 2 possible sites. The participants aged <18 years arrived at the interview with a signed parental consent form. Upon arrival at the study site, eligibility was confirmed, and consent was obtained. The consented participants completed an interviewer-administered questionnaire—administered by trained members of the study team who were South African women of reproductive age and spoke the above languages—and received 3 recruitment coupons. Participants received a participation incentive of 75 South African rand (approximately US $4) and a recruitment incentive of 50 South African rand (approximately US $2.50) for each eligible participant they successfully recruited. Participants returned to the study site to collect their recruitment incentive and completed an additional survey on their experiences participating in and recruiting for the study. Recruitment was tracked using coupon numbering.

### Ethics Approval

Ethics approval for this study was obtained from the Human Sciences Research Council Research Ethics Committee in South Africa (REC 10/18/11/15: Experiences of women who self-induce abortion in Soweto, South Africa). The amount of compensation for participation in the study was arrived at in extensive consultation with the Human Sciences Research Council. No identifying information was collected from the participants. Study-related documents, including coupons, did not disclose abortion incidence as the primary aim of the study.

### Instruments and Measurement

The main instrument in our study was a quantitative survey with questions on demographic characteristics, social network composition, health behaviors, sexual history, pregnancy history, and experience with abortion. The follow-up instrument contained questions on the recruitment process, including questions on refusals. Categories for out-of-facility providers and methods were informed by existing literature [28,30,33] as well as findings from formative research that comprised in-depth interviews conducted with 19 women from Soweto who had attempted to terminate a pregnancy outside the formal health setting [34]. In addition, once draft instruments were developed, we conducted cognitive interviews with 5 participants from the formative research phase (all of whom had consented to be recontacted) to ensure that the instruments were understandable and that the answer choices were appropriate. Minor refinements to the terminology and answer choices were made following the cognitive interview phase.

The primary outcome of interest for this study was the proportion of people who ever attempted abortion (facility-based or out-of-facility abortion), measured as the weighted proportion of women in the study who reported attempting at least 1 abortion in their lifetime. In addition, we calculated the 1-year and 5-year incidence of abortion attempts. We defined *out-of-facility* abortion as any abortion attempt, successful or unsuccessful, that did not take place under the supervision of (nor with a prescription from) a physician, nurse, or other advanced practice clinician at a government-run or privately operated health care facility. We defined facility-based abortion as any abortion attempt, successful or unsuccessful, that took place under the supervision of a physician, nurse, or other advanced practice clinician at a government-run or privately operated health care facility. The key sociodemographic variables used to compare the representativeness of our sample with the source population (women of reproductive age in Soweto) were age, educational attainment, employment status, and home language. Consistent with the RDS literature [21,35], we assessed network size using the following question: “How many women of reproductive age who live in Soweto do you know, who also know you, that you have seen in the past week?”

We additionally collected data on network characteristics such as recruiter-recruit relationships and recruitment experiences to assess whether several RDS recruitment assumptions were met in this study; full findings from this methodological assessment are published elsewhere [36].

### Statistical Analysis

Using the method proposed by Salganik [37] for calculating the desired sample size for a sample proportion, we arrived at a minimum sample size of 834 participants, which enabled us to detect a proportion of people who attempted abortion of 50% (417/834; maximally conservative estimate), with an SE <0.03 and assuming a design effect of 3. Data management was conducted using R (version 4.0.2; R Foundation for Statistical Computing) [38] and Stata (version 14; StataCorp LLC) [39]. We used RDS Analyst [40] to examine recruitment patterns, equilibrium (the point in recruitment at which the sample proportions of sociodemographic characteristics stabilize), homophily, waves of recruitment, and mean network size and compute weighted estimates of our primary outcomes. We used the RDS-II estimator to estimate the population proportions of demographic characteristics and our primary outcomes [41]. The RDS-II estimator reweights the sample population to account for *homophily,* the tendency of participants to recruit other participants who share similar characteristics [14]. Participants are weighted by the inverse of their degree (social network size); for example, participants with a degree of 10 would be given a weight of 1/10. We used imputed *visibility* for our measure of degree (effective network size), which incorporated self-reported social network size, the number of successful recruits, and the time to recruit to estimate each participant’s inclusion probability. Visibility was imputed using the *impute.visibility_mle* function in RDS Analyst [40]. We calculated 95% CIs using 1000 bootstrap replications.

## Results

### Recruitment

**Figure 1:**
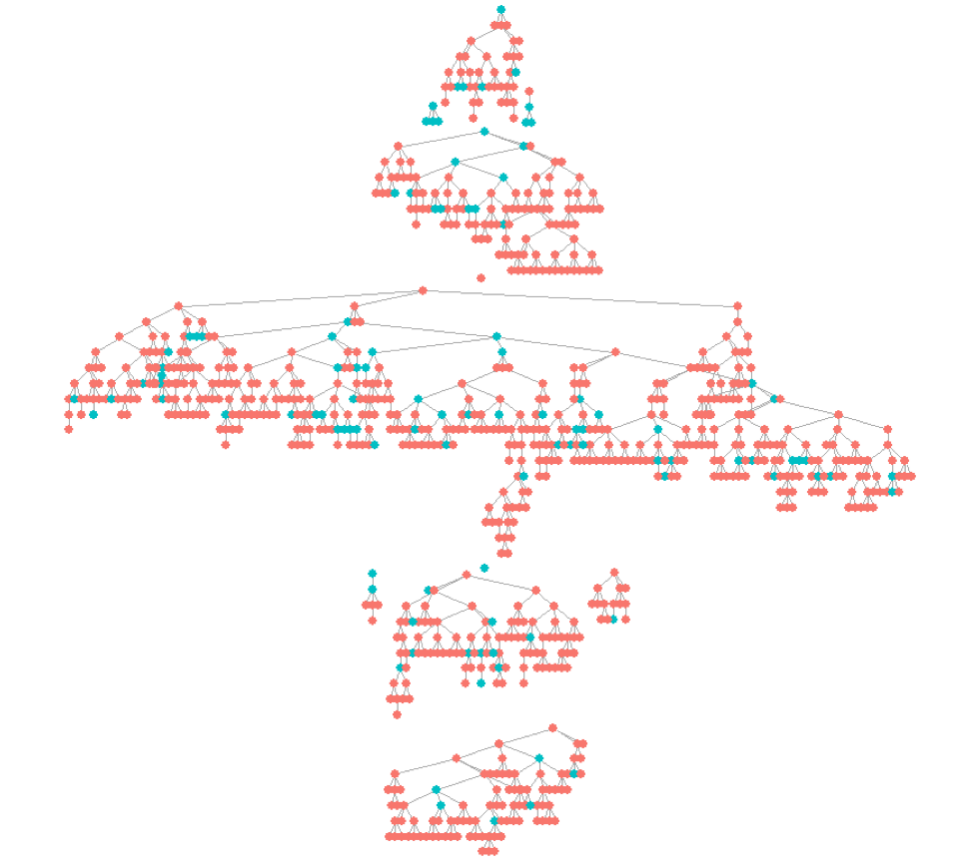
Recruitment tree from a respondent-driven sampling study of women aged 15 to 49 years in Soweto, South Africa (N=849). Each node represents a participant connected to their recruits and recruiters. Nodes in blue indicate a participant who reported any lifetime experience of abortion.

Between April 4, 2018, and December 17, 2018, 849 eligible participants were recruited into the study. Recruitment occurred over 36 weeks, and the longest recruitment chain lasted 17 waves, with a mean of 6.6 recruitment waves for active seeds. A total of 2 seeds did not recruit any participants, and 56.5% (480/849) of the sample originated from 1 seed. Approximately one-third (n=837, 36.8%) of the 2277 distributed coupons were returned. Recruitment patterns based on lifetime experience of abortion are shown in Figure 1. A methodological assessment of RDS assumptions and recruitment dynamics has been previously published [36], and the key findings are summarized below. There was strong homophily (chi-square test for independence, *P*<.05) for age, educational attainment, employment status, and lifetime experiences with abortion, suggesting a strong tendency to recruit individuals with similar characteristics to theirs as compared with random recruitment. Sample proportions for age, home language, educational attainment, and socioeconomic indicators stabilized (reached equilibrium) by approximately 300 to 500 participants, well before our estimated sample size.

### Study Population

The unweighted sample proportions for the selected demographic characteristics, along with weighted population proportions, are reported in Table 1. Table 1 presents the population estimates of the selected demographic characteristics based on publicly available data. The unweighted median age was 27 (IQR 22-36) years. Approximately one-fifth (20.2%, 95% CI 17.4%-23.1%) of the target population were currently in school, and most had at least some secondary education (52.5%, 95% CI 49.1%-56.0%) or completed secondary education (39.6%, 95% CI 36.2%-43.1%). Most were unemployed (83.1%, 95% CI 80.5%-85.8%). Although a statistical comparison of the RDS-II sociodemographic estimates to the estimated source population estimates is not possible, the sample characteristics are largely similar to the source population for all variables, except for employment status.

**Table 1:** Sociodemographic characteristics of women participating in a respondent-driven sampling survey, Soweto, South Africa, 2018 (N=849).

Sociodemographic characteristics			Sample proportion (unweighted)					RDS-II^a^ weighted proportion					Estimated proportion in source population^b^ (%)
			Participants, n (%)		95% CI		Participants (%)			95% CI			
**Age (years)**													
	15-19	133 (15.7)		13.4-18.3		16.1			14.1-18.0		13.7		
	20-24	184 (21.7)		19-24.6		22.2			19.5-24.8		18.8		
	25-29	166 (19.6)		17-22.4		19.2			17.6-20.8		19.5		
	30-34	127 (15)		12.7-17.5		14.9			12.0-17.7		15.4		
	35-39	109 (12.8)		10.7-15.3		12.6			9.8-15.5		12.6		
	40-44	78 (9.2)		7.4-11.3		9			6.6-11.4		10.6		
	45-49	52 (6.1)		4.7-8.0		6.1			3.6-8.7		9.6		
**Educational attainment**													
	Currently in school	169 (19.9)		17.4-22.8		20.2			17.4-23.1		—^c^		
	No schooling	1 (0.1)		0.0-0.8		0.1			0.1-0.2		6.8		
	Some primary	9 (1.1)		0.6-2.0		1.1			0.9-1.3		6.3		
	Completed primary	30 (3.5)		2.5-5.0		4			3.7-4.4		6.3		
	Some secondary	450 (53.1)		49.8-56.5		52.5			49.1-56.0		76.1		
	Completed secondary	335 (39.6)		36.3-42.9		39.6			36.2-43.1		76.1		
	Higher education	22 (2.6)		1.7-3.9		2.6			1.0-4.3		10.8		
**Relationship status**													
	Never married	622 (73.8)		70.7-76.6		74.1			70.8-77.3		45.6		
	Married (traditional or civil)	55 (6.5)		5.0-8.4		6.2			5.4-7.1		32.8		
	Living together	152 (18)		15.6-20.8		18			14.8-21.3		10.6		
	Divorced	9 (1.1)		0.6-2.0		1			0.7-1.4		3.1		
	Separated	5 (0.6)		0.2-1.4		0.6			0.4-0.9		0.9		
**Employment status**													
	Employed	138 (16.3)		13.9-18.9		16.9			14.2-19.6		70.6		
	Unemployed or student	710 (83.7)		81.1-86.1		83.1			80.5-85.8		29.4		
**Housing type**													
	Formal housing	676 (79.7)		76.9-82.3		79.3			76.4-82.2		81.3		
	Traditional housing	2 (0.2)		0.1-0.9		0.2			0.0-1.0		0.1		
	Informal housing	160 (18.9)		16.4-21.6		19.4			19.4-19.4		18		
	Other	10 (1.2)		0.6-2.2		1.1			0.0-4.0		0.6		
**Water source**													
	Piped water inside the dwelling	427 (50.4)		47-53.7		51.1			47.6-54.6		60.3		
	Piped water inside the yard	402 (47.4)		44.1-50.8		46.9			43.4-50.4		31.8		
	Piped water from access point outside	19 (2.2)		1.4-3.5		2			0.9-3.0		7.1		
**Toilet facilities**													
	Flush toilet (connected to sewage)	820 (96.7)		95.3-97.7		97			95.8-98.2		88.6		
	Flush toilet (with septic tank)	8 (0.9)		0.5-1.9		0.9			0.3-1.6		1.6		
	Pit toilet with ventilation	14 (1.7)		1.0-2.8		1.4			0.5-2.3		3.4		
	Pit toilet without ventilation	6 (0.7)		0.3-1.6		0.7			0.1-1.3		1.7		
**Resources**													
	Connected to electricity	838 (98.8)		97.8-99.4		99			98.9-99.1		87.5		
	Owns a television	787 (92.8)		90.8-94.4		93.3			91.5-95.2		86.5		
	Has a radio	622 (73.3)		70.2-76.2		73			70.0-76.0		70.1		
	Has a landline	80 (9.4)		7.6-11.6		9.6			7.5-11.8		—		
	Has a cellphone	841 (99.2)		98.3-99.6		99.1			99.1-99.2		95.5		
**Home language**													
	Afrikaans	2 (0.2)		0.1-0.9		0.2			0.0-3.2		1.3		
	English	4 (0.5)		0.2-1.3		0.5			0.3-0.7		2.3		
	IsiXhosa	66 (7.8)		6.2-9.8		7.7			4.4-11.1		8.7		
	IsiZulu	329 (38.9)		35.7-42.3		39.9			39.6-40.1		37.1		
	Sepedi	20 (2.4)		1.5-3.6		2.4			1.1-3.7		5.1		
	Sesotho	216 (25.6)		22.7-28.6		24.5			21.8-27.3		15.5		
	Setswana	59 (7)		5.4-8.9		7.2			4.8-9.6		12.9		
	Tshivenda	35 (4.1)		3.0-5.7		4			2.0-6.0		4.5		
	Xitsonga	100 (11.8)		9.8-14.2		12.1			11.8-12.5		8.9		
	Shona	13 (1.5)		0.9-2.6		1.3			1.0-1.6		0		
	Other	1 (0.1)		0-0.8		0.1			0.0-2.0		3.7		

^a^RDS-II: respondent-driven sampling II.

^b^Source population proportion estimates are from the 2016 South Africa Community Survey, localized to Johannesburg for age, educational attainment, housing type, water source, toilet type, and electricity access. Data on relationship status and other resources were obtained from the 2016 South Africa Community Survey, localized to Gauteng Province. Data on employment status are from the Labor Force Survey and from the Quarterly Labour Force Survey published by Statistics South Africa; data represent unemployment rates among women in South Africa from July 2018 to September 2018.

^c^Census data not available for comparison.

### Abortion Attempts

The RDS-II estimates of the proportion of people who ever attempted abortion was 12.1% (95% CI 9.7%-14.4%; Table 2). A total of 7.1% (95% CI 5.4%-8.9%) reported a facility-based abortion, and 4.4% (95% CI 3.0%-5.8%) reported an out-of-facility abortion. The true design effect for the main outcome, any abortion attempt, was 1.14. Most participants (RDS-II estimate: 61.8%, 95% CI 58.4%-65.2%) reported that their best friend had an abortion (not displayed in the tables).

Because of likely underreporting, we present the unweighted proportions for various abortion experiences. A total of 106 out of 849 (12.5%) participants reported at least one abortion attempt at any point in their lifetime, and 9 (n=106, 8.5%) of them did not provide any subsequent information about their experiences. Among the remaining 97 (91.5%) participants who reported an abortion attempt and answered additional questions related to their abortion experience, 85 (88%) attempted an abortion once, 8 (8%) reported 2 abortion attempts, and 4 (4%) reported ≥3 abortion attempts. When asked about their most recent abortion attempt, 60 (62%) participants reported that they went to a health care facility. Among those who went to a health care facility, 9 (15%) attempted to do something on their own to end their pregnancy before seeking facility-based health care, most commonly taking a laxative. At the health care facility, 28 out of 60 (47%) participants reported taking medications for abortion, 10 (17%) participants reported a surgical procedure, and 2 (3%) participants did not know what method was used to end their pregnancy. Of the participants who received medications (n=28), 2 (7%) did not have a complete abortion and continued with their pregnancy. Of the participants who went to a health care facility (n=60), 17 (28%) did not ultimately end up having an abortion because they decided that they wanted to continue with their pregnancy, they were counseled to continue with the pregnancy, or their gestational age was beyond the legal limit.

Of the 97 participants, 37 (38%) of the participants who did not report going to a health care facility for their most recent abortion attempt reported using methods such as laxatives, aspirin, strong tea or coffee, pesticides, bleach, or combinations of the above. Of these 37 participants, 22 (59%) reported successfully terminating their pregnancy.

**Table 2:** Proportion of ever attempting abortion, 1-year incidence of abortion, and 5-year incidence of abortion in a respondent-driven sampling survey, Soweto, South Africa, 2018 (N=849).

Lifetime experience of abortion			Unweighted estimate						RDS-II^a^ estimate						Design effect
			Participants, N^b^		Participants, n (%)		95% CI		Participants (%)			95% CI			
**Abortion attempt**															
	People who attempted to have an abortion	849		106 (12.49)		10.4-14.9		12.1			9.7-14.4			1.14	
	People who attempted to have an in-facility abortion^c^	841		63 (7.49)		5.9-9.5		7.1			5.4-8.9			1.05	
	People who attempted to have an out-of-facility abortion^c^	841		37 (4.3)		3.2-6.0		4.4			3.0-5.8			1.03	
**Incidence of abortion^d,e^**															
	1-year incidence of abortion attempts (2017)	840		9 (10.71)		3.74-17.70		9.14		1.90-16.40			1.18		
	5-year incidence of abortion attempts (2013-2017)	840		42 (50)		33.82-66.18		47.3		35.64-58.96			0.54		

^a^RDS-II: respondent-driven sampling II.

^b^The total sample of 849 participants denotes those who attempted to have an abortion. The location of abortion attempt is missing for 8 participants; therefore, the N value is smaller for the location rows. The data are missing for the year of abortion for 9 participants; therefore, the N value for incidence is 840.

^c^Data on the type of abortion (in vs out of facility) are missing for 8 participants.

^d^Data on the year of abortion are missing for 9 participants.

^e^Incidence of abortion per 1000 women.

## Discussion

### Principal Findings

In our study, we explored the feasibility of applying the RDS methodology to estimate the proportion of women of reproductive age who have ever attempted abortion in Soweto, South Africa. The estimated proportion of ever attempting an abortion of 12% and 1-year incidence of 9.1 abortion attempts per 1000 women of reproductive age in our study likely represents a substantial underestimation of the actual abortion experiences in this study population [27]. Although no directly comparable measures exist, recently published, country-specific estimates of abortion incidence report an annual estimated 30 abortions occur per 1000 women of reproductive age in South Africa, representing a figure nearly 3 times the magnitude of the comparable estimate from our study [42]. We posit that this underestimation represents a failure of the RDS method to generate more reliable estimates of the abortion incidence in our study.

We previously published a methodological assessment of whether several RDS assumptions were met in these data [36]. In that paper, we found that although the approximation of sampling with replacement was met, the participants did not consistently report the same degree, nor did they randomly recruit from within their social network. It is likely that the failure to meet the assumptions yielded a sample with different employment characteristics than those of the target population, which was not resolved by standard RDS methods. However, without gold standard abortion estimates for the target population by sociodemographic characteristics, it is challenging to assess the impact of failing to meet these assumptions on inference for abortion. Although it is plausible that some of the underestimation of abortion may have been due to the overrepresentation of unemployed participants in the sample, it is more likely to have been due to underreporting.

### Strengths and Limitations

This study highlights the limitations of RDS in measuring abortion. Although the social networking literature is lacking on the subject of abortion, public health evidence suggests that those who have abortions outside the formal health sector communicate with members of their social network to obtain information about self-managed or community-based abortions [43-45]. We hypothesized that RDS could offer a previously untested alternative method to more accurately measure abortion incidence by similarly relying on peer recruitment to help reduce the underreporting of abortion. However, it is possible that because we recruited from a general population of women of reproductive age, peer recruitment operated in the opposite direction in our study; if participants were recruited into the study by members of their social network who they would not want to know about their prior abortions, they might have been less likely to report their abortions to the researchers conducting the study. In this context, it is notable that most participants in our study reported that their best friend had had an abortion—potentially indicating a willingness, as seen in other studies, to discuss the abortion experiences of others but not themselves. In addition, as we lack representative sociodemographic data on reproductive-aged women localized to Soweto, we were unable to directly validate whether RDS sampling generated a sample with demographic characteristics similar to those of the overall population of women of reproductive age living in Soweto. However, based on the best available estimates (population-based data from Gauteng Province and the city of Johannesburg, where Soweto is located), our sample differs substantially from the source population, particularly with respect to employment, even after adjusting using RDS inference procedures.

However, the limitations of our study should be considered in the context of the strengths. We successfully recruited a large sample of women of reproductive age, demonstrating that RDS can be used to recruit a sample of participants who are willing to participate in a study about reproductive health, answer questions about abortion, and participate in peer recruitment. We hope that the lessons learned from our study will be instructive to future researchers exploring the use of novel sampling approaches for measuring abortion.

Despite the failure of RDS to generate more reliable estimates of abortion incidence, it may be a method best suited for sampling when selecting on stigmatizing characteristics (injection drug use, men having sex with men, sex work, and now, abortion), as it has most commonly been applied. For example, RDS could be used to sample a population with out-of-facility abortion experience and estimate the proportion of that population that has experienced one or more outcome of interest (eg, using medication abortion, seeking health care in the formal health sector, or experiencing complications). Other population size estimation methods could be deployed to arrive at estimates of prevalence in an RDS study that is specific to abortion experiences.

It is conceivable that RDS could reduce underreporting of abortion if it were deployed to estimate abortion incidence among a highly socially networked population (potentially in humanitarian settings, among sex workers, etc). However, it is also possible that asking questions about stigmatizing experiences in any general population–based survey will be subject to underreporting—especially when interviews are administered face-to-face. Using tools such as Audio Computer Assisted Self-Interviewing and other technologies has been shown to reduce underreporting in studies of some stigmatized behaviors and could prove useful in the context of abortion as well [46,47].

### Conclusions

Accurate estimates of abortion incidence within and outside formal health settings are vital for developing targeted and effective programs, policies, and interventions to increase the access to safe abortions. In certain highly networked populations, RDS may prove to be a useful tool in the toolkit of abortion researchers, but to ensure that people seeking abortion have the information and support they need, regardless of where or how their abortion takes place, more work is needed to develop and validate tools that more accurately measure not only the incidence of abortion but also the experiences, quality, and outcomes of abortions.

## Abbreviations

RDS: respondent-driven sampling
